# Increased Cell Proliferation and Mucocyte Density in the Sea Anemone *Aiptasia pallida* Recovering from Bleaching

**DOI:** 10.1371/journal.pone.0065015

**Published:** 2013-05-28

**Authors:** David Fransolet, Stéphane Roberty, Anne-Catherine Herman, Linda Tonk, Ove Hoegh-Guldberg, Jean-Christophe Plumier

**Affiliations:** 1 Ecophysiologie Animale, Université de Liège, Liège, Belgium; 2 Laboratoire d’écologie animale et d’écotoxicologie, Université de Liège, Liège, Belgium; 3 ARC Centre of Excellence for Coral Reef Studies and School of Biological Sciences, The University of Queensland, St. Lucia, Queensland, Australia; 4 Global Change Institute, The University of Queensland, St. Lucia, Queensland, Australia; Institut National de la Recherche Agronomique (INRA), France

## Abstract

Recovery of coral after bleaching episodes is a critical period for the health of the reef ecosystem. While events such as symbiont (genus *Symbiodinium*) shifting/shuffling or tissue apoptosis have been demonstrated to occur following bleaching, little is known concerning tissue recovery or cell proliferation. Here, we studied the sea anemone *Aiptasia pallida* exposed to a transient elevation of water temperature combined with high illumination (33°C and 1900 µmolphotons.m^−2^.s^−1^ for 30h). Following such treatment bleached anemones showed a significant reduction of their *Symbiodinium* density. Cell proliferation in the ectodermis and gastrodermis was determined by assessing the densities of cells labeled with a thymidine analogue (EdU). Cell proliferation significantly increased during the first day following stress in both tissue types. This increased cell proliferation returned to pre-stress values after one week. Although cell proliferation was higher in the ectodermis in absence of stress, it was relatively more pronounced in the gastrodermis of stressed anemones. In addition, the ratio of ectodermal mucocytes significantly increased three weeks after induced stress. These results suggest that thermal/photic stress coupled with the loss of the symbionts is able to enhance cell proliferation in both gastrodermis and ectodermis of cnidarians. While new cells formed in the gastrodermis are likely to host new *Symbiodinium*, the fate of new cells in the ectodermis was only partially revealed. Some new ectodermal cells may, in part, contribute to the increased number of mucocytes which could eventually help strengthen the heterotrophic state until restoration of the symbiosis.

## Introduction

High sea surface temperature (SST) accompanied by high levels of solar irradiance are known to disrupt the symbiosis between scleractinian corals and endosymbiotic dinoflagellates of the genus *Symbiodinium* (aka coral bleaching) Studies have shown that these environmental factors can act both separately [1]–[6] and in combination [7]–[11]. Coral bleaching typically involves an impairment of algal photosynthesis and eventually loss of the algae from the host tissue [12]–[15] and therefore deprives the host of its main energy source causing a disruption of symbiosis [16]. Consequently, during the weeks following this disruption the nutritional state of the coral is compromised. Depending on the symbiont/host association and the intensity of the stress, the coral could either die [17]–[19] or survive [1], [20], [21] through a process of recovery that until now is still poorly characterized.

Cases of coral recovery have highlighted many cellular modifications occurring in the host tissue of the energetically compromised coral. A number of studies have come to conflicting conclusions about the role of mucocytes and mucus secretion in particular. Some hypothesize that mucus production is dependent on *Symbiodinium* for energy supply and carbon input [22], [23]. Indeed a reduction of *Symbiodinium* cell density was induced by shading, eliciting a decrease in mucus production [23], [24]. Other studies have reported increases in mucus release [22], [25], [26] or mucocyte number [25], [27] following bleaching. This variation can be partially explained by inter-specific variation. For example, Lasker et al. [25] showed that mucocyte number could increase or decrease after bleaching depending on the coral species involved.

The mucus has critical functions for coral protection and feeding [28]–[30] but also plays a fundamental role of energy carrier in reef ecology [31], [32]. It contains antimicrobial substances controlling the associated microbial community [33] and it stimulates planktonic or benthic microbial activity [31], [34]. Moreover, owing to its adhesive character, attached and/or secreted coral mucus acts as a particle trap and accumulates suspended inorganic and organic particles from the water column, thus supporting the retention and the recycling of essential nutrients within the reef ([31]; see Bythell and Wild [32] for review). Therefore, modifications of the mucus production caused by environmental stress factors related to climate change could have dramatic consequences in organic matter recycling and have the potential to affect the coral reef ecosystem [33], [35].

Although observations concerning mucocytes are important, post-bleaching recovery in hermatypic cnidarians is primarily characterized by the return of pigmentation due to the symbionts within the host tissue. While bleaching can be attributed to a decrease in chlorophyll concentration it most often implies the loss of algae from host cells [36]. Many studies have focused on the mechanisms involved in *Symbiodinium* loss [37]. Several scenarios like symbiont digestion via autophagy [38], [39], symbiont expulsion [40], [41] and host cell detachment [42]–[44] have been considered [11]. However, most recent findings argue for mechanisms implying the death of host cells either by necrosis or apoptosis [19], [45]–[48]. These apoptotic pathways induced by bleaching events were first reported in the zooxanthelate sea anemone *Aiptasia pallida* [46] which is often used as a model cnidarian [49]–[51].

Several studies have documented the recovery of corals from bleaching [1], [20], [21], highlighting eventual modifications in the algal community of the host [1], [20], [36], [52]–[54]. Although understanding of re-infection mechanisms of healed host tissue is progressing [55] little is known concerning tissular mending [36] or regeneration processes that occur during this recovery. Most knowledge on tissue regeneration comes from studies on *Hydra*. In that model organism, tissue regeneration requires the cooperation of three stem cell populations: ectodermal epithelial stem cells, endodermal epithelial stem cells and interstitial stem cells. The latter provides cells committed to specific differentiation pathways leading to one class of somatic cells: neurons, nematocysts and secretory cells (of which gland cells are only detected in the body column and mucus cells in the head region), (for review see Bode [56] and Galliot and Ghila [57]). In *Hydra* mucus cells are replaced by at least two mechanisms: 1) proliferation of interstitial stem cells followed by their differentiation, and 2) transdifferentiation (no cell division) of gland cells of the body column combined with a translocation to the head region [56], [57]. Some of these pathways and mechanisms are generalized as tissue regeneration of sea anemones. However strong differences exist between these model organisms, among those is the presence of mucus cells in the tentacles of *A. pallida* but not in *Hydra* [56], [57].

In the present study we try to clarify some of the histological modifications induced by bleaching stress in cnidarians. We hypothesize that new host cells rapidly replace cells lost during bleaching in order to regenerate the damaged gastrodermis. To address our hypothesis we investigate the cellular proliferation following bleaching stress (high temperature combined with high irradiance) in the zooxanthelate sea anemone *Aiptasia pallida*. Our results provide insight in changes occurring in the gastrodermis and ectodermis as well as mucocyte dynamics following thermal stress and bleaching, with important potential insights into the response of reef-building corals to similar challenges.

## Materials and Methods

### Biological material

*Aiptasia pallida* specimens were collected in the public aquarium of the University of Liège. Individuals were kept in artificial sea water (Reef Crystals, Aquatic systems, France) for several weeks providing a multi-clonal population of anemones. Light was provided on a daily cycle of 12h/day at an intensity of 30–50 µmol photons m^−2^ s^−1^. The temperature in the aquaria was electronically controlled with a Dupla T-Control Delta (Dohse Aquaristik, Germany) in combination with a cooling unit (Titan 150, Aqua Medic, Germany) to ensure a constant temperature of 26±0.2°C. *A. pallida* were fed weekly with frozen *Artemia* shrimps, except during experiments.

### Induction of bleaching by thermal/photic stress

Twenty-four hours before the beginning of the experiment, sea anemones were placed in Petri dishes in which the water was constantly renewed by a flow-through mechanism using a peristaltic pump. Anemones were maintained at control conditions or subjected to a stress treatment (adapted from Bhagooli and Hidaka [58]). The stress treatment consisted of a 30h exposure to 33°C and illumination of approximately 1900 µmol photons.m^−2^.s^−1^ (measured in the Petri dishes using a Submersible Spherical Micro Quantum Sensor (Walz, Germany) connected to a LI-250A Light Meter (Li-Cor, USA)) produced by led bulbs (12W, 6000K, Elix Belgium). Such light intensity has previously been detected in the field [59] and the combination of temperature and high irradiance is widely used to induce loss of symbionts or bleaching in cnidarian hosts [60], [61]. All anemones were then returned to delimited parts of the same experimental aquarium and allowed to recover under normal conditions (see Biological material). The first group of anemones (one day post-stress group) was incubated immediately after the stress in EdU-containing sea water for 24 hours, a second group (one week post-stress group) was incubated for 24 hours in EdU-containing sea water at the 6th day after the end of the stress, a third group at the 20^th^ day (three weeks post-stress group) and the last group at the 55^th^ day (eight weeks post-stress group). Anemones that were not subjected to stress were sampled and incubated in EdU-containing sea water for 24h at the same time points as the stressed groups (1 day, 1 week, 3 weeks and 8 weeks post-stress) and served as controls (N = 10–17/time point). In addition, anemones (pre-stress group) were also sampled and incubated in EdU-containing sea water before stress conditions commenced. Sampling was performed at the same time of the day for each group.

### *Symbiodinium* identification and population density

The dominant *Symbiodinium* type from our pool of *A. pallida* was identified as a clade B1 by denaturing gradient gel electrophoresis (DGGE) and sequencing of the internal transcribed spacer region 2 of the ribosomal DNA (ITS2 rDNA). Following DNA extraction with a DNeasy Plant Mini Kit (Qiagen, Netherlands), the ITS2 rDNA region was amplified using the forward primer ‘ITSintfor 2′ (5′-GAATTGCAGAACTCCGTG-3′) and a reversed primer with a GC-clamp ‘ITS 2 clamp’ (5′-CGCCCGCCGCGCCCCGCGCCCGTCCCGCCGCCC CCGCCCGGGATCCATATGCTTAAGTTCAGCGGGT-3′) producing a fragment size of 330–360 bp [62]. Amplification products were screened for polymorphisms using DGGE (Biorad DCode system) and run on acrylamide gels (30–65% gradient) following the manufacturer’s instructions (Biorad Laboratories). Dominant bands were excised, re-amplified and subsequently sequenced at the Australian Genome Research Facility University of Queensland, Australia) using an ABI 3730x/sequencer in combination with BigDye Terminator sequencing reaction kits. Sequences were then examined using Codoncode Aligner version 3.5.3. (Codoncode Corporation) and identified by BLAST comparisons in GenBank.

Bleaching was estimated in each set of experimental groups using a coral health chart, as usually done on coral reefs to grossly assess the health of coral colonies during diving. In addition, to confirm that bleaching resulted from a loss of *Symbiodinium* cells as previously described in *Aiptasia sp.* [46] we evaluated *Symbiodinium* density in tentacle sections of the pre-stress and bleached anemones during the recovery period (N = 3/time point).

### Tissue histology

Histological techniques were used to evaluate the cellular proliferation and the number of mucocytes in control and bleached anemones isolated at each time point (N = 10–17/time point). Cell proliferation assays consisted of counting nuclei which incorporated thymidine analogue during DNA synthesis. To do so, each anemone was incubated for 24h in a solution of 1 µM EdU (5-ethynyl-2′-deoxyuridine, thymidine analogue, Invitrogen, Eugene-Oregon-USA) in seawater [62]. Anemones were then anesthetized for 15 minutes in a 1∶1 solution of seawater and 0.37 M MgCl_2_ before fixation in a solution of 4% paraformaldehyde in seawater. Fixed specimens were subsequently dehydrated, embedded in paraffin (paraplastXtra, Sigma), cut into 5 µm thick slices and finally placed on silane-coated slides. After dissolution of the paraffin and re-hydration the slides were washed three times for 5 minutes in Phosphate-Buffered Saline (PBS; 3.82 g/L NaH_2_PO_4_.2H_2_O; 10.48 g/L Na_2_HPO_4_ in 0.45 M NaCl). Then the slides were incubated for 10 minutes in a blocking solution of 3% Bovine Serum albumin in PBS in order to prevent non-specific interactions. This was followed by a permeabilization procedure of 20 minutes in a solution of 0.5% Triton x-100 in PBS prior to three PBS washes for 5 minutes and incubation for 30 minutes in the reaction mix made from the Click-iT EdU kit (Click-iT EdUAlexa Fluor 488 Imaging Kit, Invitrogen, Eugene, Oregon, USA). After 3 washes for 5 minutes in PBS the slides were incubated for 15 minutes in a 5 µM solution of WGA (wheat germ agglutinin + Alexa 594, Invitrogen, Eugene-Oregon-USA) in order to label the mucocytes [23]. Finally, the slides were washed 3 times for 5 minutes in PBS, dried and mounted for microscopy (Vectashield + DAPI, Vektor labs, Burlingame CA, USA). Slides were examined under a fluorescence microscope (Nikon TE2000-U). Omission of Clik-iT solution during the revelation step and detection of fluorescence in anemones that were not incubated in EdU were used to verify the specificity of fluorescent signals. Some sections were also observed following standard hematoxylin/eosin staining procedures to visualize *Symbiodinium* cells using transmitted light microscopy.

### Analyses and Statistics

Mean densities of *Symbiodinium*, EdU-positive nuclei and mucocytes were calculated from five counts made in randomly sampled tentacle sections of each anemone using Nikon NIS software v3.1. The numbers of *Symbiodinium*, EdU-positive nuclei and mucocytes were reported to the tissue area [23], [26], [29]. The ectodermal area, rather than the entire tissue area, was used to standardize counts because bleaching is known to affect the gastrodermis due to algal loss and cell death [46]. Counting the total number of ectodermal nuclei was not possible because nuclei were too tightly packed to distinguish individual nuclei. At each time point, cell proliferation was obtained by dividing the density of EdU+nuclei of stressed anemones by that of control anemones. Statistical analyses were performed using Statistica v10. *Symbiodinum* densities were analyzed at each time point using Student t-test. Analyses of variance (one-way ANOVA) followed by Dunnett’s *post hoc* test were used to compare ratios of cell proliferation and mucocyte densities after stress to pre-stress ratio.

## Results

### Population density of *Symbiodinium*

**Figure 1:**
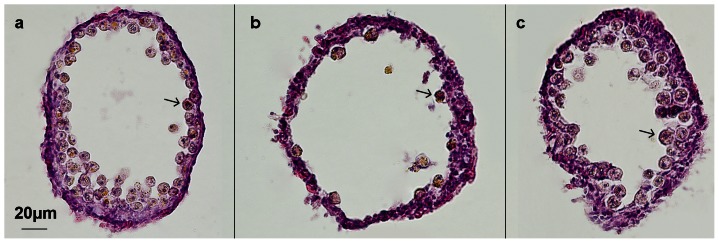
Transient reduction of *Symbiodinium* density following bleaching. H&E stained transversal sections of tentacles illustrating *Symbiodinium* (*arrows*) density in the gastrodermis of anemones before the bleaching procedure (**a**) and after 1 week (**b**) and 8 weeks (**c**) of recovery. After 8 weeks the gastrodermis has regained its normal appearance.

**Figure 2:**
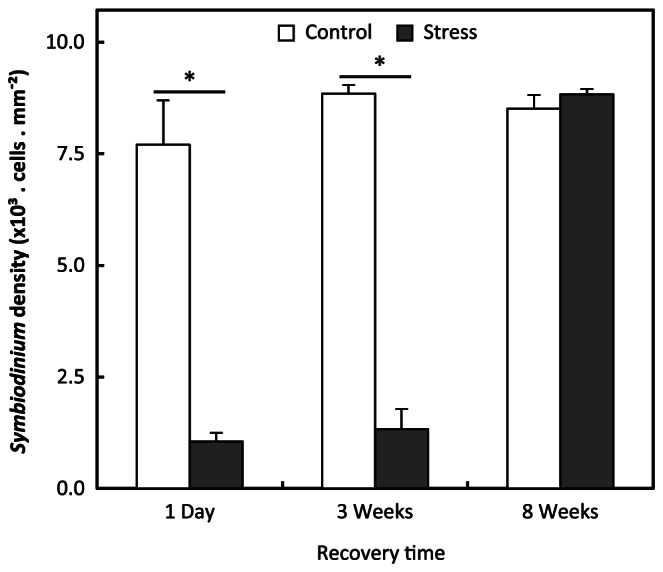
Loss of *Symbiodinium* following photic/thermic stress. *Symbiodinium* density (mean ± S.E.M.) was lower in stressed anemones than in controls 24h hours and 3 weeks after the stress. No difference between groups was detected eight weeks after stress. Asterisks represent values significantly different than controls (Student *t* test; p<0.05).

The light and temperature treatment successfully bleached anemones as observed by evaluating the color of anemones before and after the induced stress using the coral health chart (coral watch) as reference. Similarly variations of algal densities after stress were also detected in hematoxylin/eosin stained sections of tentacles (Fig. 1). A quantitative confirmation that bleaching was caused by loss of algae was obtained by measuring *Symbiodinium* density before and after stress (Fig. 2). Control anemones showed a similar density (about 8×10^3^ algae per mm^2^ of ectodermal area; mean ± SEM) at each time point after photic/thermic stress. Twenty four hours after the stress, anemones of the stress group showed a density (1.1±0.2×10^3^ algae.mm^−2^) significantly lower than controls (7.7±1.0×10^3^ algae. mm^−2^; Student *t* Test; p = 0.0003). *Symbiodinium* densities were still lower (p = 0.00001) in stressed anemones three weeks after the stress ended (1.3±0.5×10^3^ and 8.9±0.2×10^3^ algae. mm^−2^, respectively). *Symbiodinium* densities were similar in control and stress groups eight weeks after the end of the stress (Fig. 2) with a density of 8.5±0.3×10^3^ and of 8.8±0.1×10^3^ algae. mm^−2^, respectively (p = 0.16).

### Proliferation of cells within ectoderm and gastrodermal tissues

Histological analyses revealed that EdU was detected in tentacle tissues. Histological controls confirmed that fluorescent signals corresponded to EdU labeling in tentacle tissues and not to cellular autofluorescence or methodological artefacts due to the protocol of EdU revelation.

**Figure 3:**
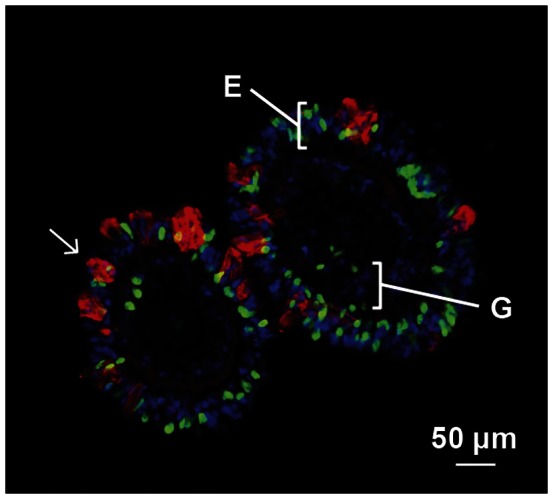
EdU and WGA labeling. Transversal section of a tentacle showing histological labeling of EdU^+^ nuclei (*green*) and mucocytes (*arrow*) stained with WGA (*red*). DAPI staining (*blue*) was used to visualize nuclei. E, endodermis; G, gastrodermis.

Under normal conditions (i.e., in anemones of the pre-stress group) EdU-positive cells were observed in both the gastrodermis and the ectodermis. However, the number of EdU+ cells strongly differed between these tissues (Fig. 3). The number of EdU+ cells in the ectodermis of control anemones was about 16-fold higher than in their gastrodermis. Similarly, in stressed anemones, higher numbers of EdU+ cells were also observed in the ectodermis compared to the gastrodermis.

**Figure 4:**
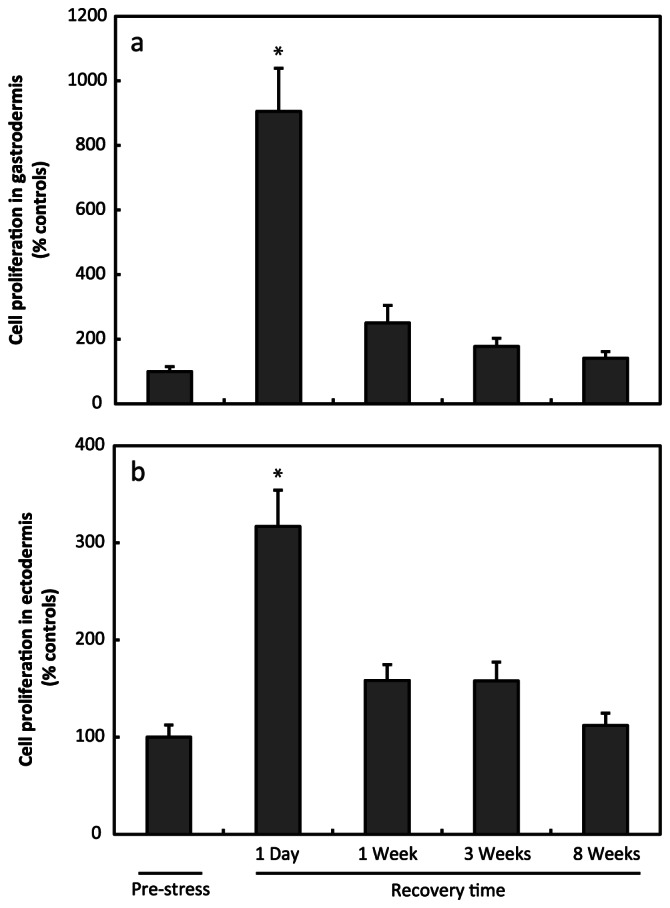
Increase of cell proliferation after bleaching. Cell proliferation (mean ± S.E.M.; EdU+ cell density in treated anemones divided by EdU+ cell density in controls) in the gastrodermis (**a**) and ectodermis (**b**) shows a rapid and transient increase following the bleaching procedure (N = 10–17/time point). Asterisk represents values significantly different than pre-stress values following ANOVA and Dunnett’s *post hoc* test (p<0.001).

To assess the effects of stress on cell renewal in the gastrodermis and the ectodermis we determined the ratio of EdU+ cell densities between stressed and control tissues at each time point. In the gastrodermis of bleached anemones, a rapid increase of cell proliferation ratio was observed following the stress period (Fig. 4a). During the first day following the bleaching stress cell proliferation increased to 905±133% of controls (mean ± SEM). After one week, the ratio was down to 250±54% and remained low at three weeks and eight weeks after stress (177±26 and 141±21% of controls, respectively). ANOVA (F(4,62)  = 21.350) followed by Dunnett’s *post hoc* test confirmed that cell proliferation ratio was significantly higher immediately after stress, at the beginning of the recovery phase than before stress (p<0.001). In the ectodermis, we observed the same trend as in the gastrodermis, that is, a transient increase in cell proliferation ratios after stress (Fig. 4b). Immediately after the stress was induced the cell proliferation (317±38% of controls) was slightly higher than when measured before stress. One week, three weeks and eight weeks after stress the ratios (158±16, 158±20 and 112±13% of controls, respectively) were close to those measured in anemones of the pre-stress group. Statistical analyses revealed that cell proliferation in the one day post-stress group was significantly higher than in the pre-stress group (F(4,62)  = 14.628; Dunnett’s *post hoc* test, p<0.0001).

### Mucocytes

**Figure 5:**
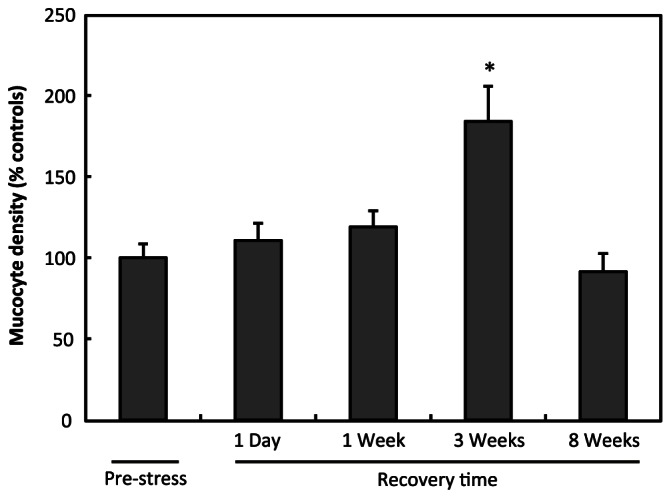
Increase of mucocyte density after bleaching. Mucocyte density (mean ± S.E.M.) in the ectodermis shows a delayed and transient increase following the bleaching procedure (N = 10–17/time point). Asterisk represents values significantly different than pre-stress values following ANOVA and Dunnett’s *post hoc* test (p<0.0001).

Although the number of ectodermal mucocytes varied between batches of anemones used for each experiment (73±27 to 437±154 cells.mm^−2^), it remained similar in all control anemones (controls of the pre-stress group as well as controls of one day, one week, three weeks and eight weeks post stress groups) within a given experiment. The ratios of mucocyte densities were similar in the ectodermis of anemones before stress, one day and one week after stress. However the ratio was higher three weeks after the end of the bleaching stress (184±22% of controls; F(4,60)  = 7.822; p<0.0001). No significant difference was observed eight weeks after stress (Fig. 5).

## Discussion

We explored an important step in tissue regeneration occurring in cnidarians following an exposure to high temperature and irradiance leading to a transient disruption of symbiosis with *Symbiodinium* algae. We focused on histological modifications taking place in *Aiptasia pallida* during this recovery period.

Recent studies on mechanisms involved in the loss of symbiotic dinoflagellates during coral bleaching highlighted that this loss is potentially related to loss of gastrodermal host cells, leaving the gastrodermis heavily damaged [42], [44]. Here we show that cellular proliferation was significantly enhanced in the gastrodermis following bleaching in the sea anemone *Aiptasia pallida*. This suggests that a massive cellular proliferation rapidly occurs in response to stress. The exact timing of initiation of this response is uncertain. Cell proliferation could be triggered at the beginning, during or at the end of the induced stress, when conditions return to normal. In any case cell proliferation returned to normal levels one week after the induced stress, suggesting that the gastrodermis has recovered from its stress induced cell loss. Indeed, a large part of the gastrodermis can remain healthy, despite the bleached state of anemones [46]. Therefore, a normal level of cell proliferation may suffice to rebuild the gastrodermal cell layer with time. Studies of cell turnover in the gastrodermis may clarify this suggestion.

Cell proliferation increased immediately following bleaching in stressed anemones, most likely in order to regenerate the damaged tissue and eventually regain the symbiotic state with algae. New *Symbiodinium* cells in recovering anemones were sourced from the proliferation of those that remained in the bleached hosts or were recently expelled from them as the seawater used in our experiments was artificial, regularly renewed and thus lacked any live *Symbiodinium*. These observations complement results of other studies reporting on the loss of host cells during bleaching [19], [45], [46] and suggest that gastrodermal regeneration may represent an important step in the recovery process of bleached cnidarians.

Surprisingly, our results also show an increase in cell proliferation in the ectodermis of bleached anemones. Although this increase is less striking than that observed in the gastrodermis, with values 3 times higher in the ectodermis one day after bleaching stress than in the control experiment, this observation was unexpected because the ectodermis of *A. pallida* is reported to suffer only little damage following bleaching [46]. Cell proliferation in the ectodermis is generally relatively high, even under normal conditions, suggesting a large potential to recover from the relatively limited damage induced by stress. It is therefore unlikely that our observations are solely related to regeneration processes. A plausible additional explanation would be an augmentation in the production of cellular phenotypes that potentially improve the survival of the bleached host. Here we focus on mucocytes, a cell type that has often been reported to be crucial for the holobiont (host animal and symbionts) [32], but whose response to bleaching is not yet completely understood and still subject to debate [23], [27].

Although an increase in mucus release has been well documented in stressed corals following various environmental stressors, including heat stress and high irradiance [63] changes in the population density of mucocytes in bleached organisms remain unclear. Lasker et al. [25] found an augmentation of epidermal mucocyte cells in bleached samples of *Favia fagrum*. Glynn et al. [27] observed divergent results in bleached corals. Bleached samples of *Pavona clavus* presented an increase in mucous secretory cells of the epidermis but samples of *P. gigantea* and *P. varians* had fewer mucous secretory cells compared to the healthy samples. More recently Piggot et al. [23] reported that the number of epidermal mucocytes in *Montastraea annularis* diminished when shading increased and, conversely, was higher in samples obtained during a seasonal increase in sea surface temperature. Both conditions resulted in a reduction of algal density. All these observations suggest that factors, such as host species identity and the nature of the stress, may influence the number of epidermal mucocytes.

We found that mucocyte densities range between 70 to 440 cells.mm^−2^ in *A. pallida*. These values seem relatively consistent with mucocyte density reported in the epidermis of coral species, ranging from 220 cells.mm^−2^ to 3,000 cells.mm^−2^ depending on the host species and light conditions [23], [29]. The coral *Mycetophyllia reesi* harbors about 3,000 mucocytes.mm^−2^ of epidermis [29] while *Montastraea annularis* showed seasonal variation of mucocytes densities from 220 cells.mm^−2^ during spring to 1,750 cells.mm^−2^ during summer [23]. We found that epidermal mucocyte density was slightly low in the sea anemone *Aiptasia pallida* in absence of stress. This density may be explained by intrinsic differences between sea anemones and coral species or by the low level of light intensity used in culture (30–50 µmol photons m^−2^ s^−1^) [23].

The mucocyte was affected by a combination of hyperthermia and increased irradiance. We did not observe a decrease in mucocyte density, which is expected if the host is energetically impaired immediately following stress. It is possible that such changes occurred during the induced stress and that by sampling one day after the stress such reduction in mucocyte density was overlooked if recovery was rapid. However, we observed a significant and transient augmentation of mucocyte density in the ectodermis 3 weeks after bleaching. The lag period observed between the cell proliferation peak and mucocyte density peak could account for the time needed to produce mature mucocytes, that may even have been produced outside of the tentacle as secretory cell precursors as seen in *Hydra* [64]. Another plausible and non-exclusive explanation for this delay is that differentiation of new cells into mucocytes are only engaged after a certain threshold (depletion of lipid stores [65] or other physiological signals [63]) that was not yet reached one week after bleaching induction.

By producing and secreting mucus, mucocytes contribute to important roles in the holobiont such as: UV protection, microbial defense, sediment cleansing, energy carrying and particle trapping [22], [28]–[30], [32], [65]–[67]. In the bleached anemone, the ability of mucus to trap particles and carry them to the hosts’ mouth is highly profitable. Therefore, even if the host is energetically impaired, increased production of mucocytes and mucus are in fact a helpful strategy. Heterotrophic feeding can sustain the hosts’ energy incomes and compensate for a reduction of algal autotrophic contribution [22], [30], [68]. This idea is corroborated by a recently developed model in which autotrophy significantly offsets effects of bleaching principally by restoring lipid stores inside host cells [65]. Augmentation of mucus production as such, potentially reflects a strategy to limit photoinhibition in algae and subsequent production of oxidative radicals [63] or help to protect the bleached, and thus more susceptible, host against UV radiation or pathogens [22], [30]. Conversely, when stressed anemones have recovered *Symbiodinium* densities similar to controls (at eight weeks after stress), mucocyte densities in stressed anemones were also similar to controls, arguing for a relationship between mucocyte and *Symbiodinium* densities in *Aiptasia*.

Mucocytes are not the only cell type derived from the increased proliferation in the ectodermis. Indeed, the density of EdU+ cells was several fold higher than the density of mucocytes (see Fig. 3). Among those EdU+ cells, a small number will differentiate and mature in mucocytes. Some of those newly produced ectodermal cells may differentiate into other cell types such as cnidocytes (aka nematocysts or stinging cells). Increased differentiation into cnidocytes is very likely in bleached anemones considering their major role in heterotrophic feeding [69]. Some of the EdU+ cells may also migrate to the gastrodermis. Indeed, in *Hydra*, ectodermal cells committed to secretory cell lineages can migrate through mesoglea to the gastrodermis [56]. If similar mechanisms are present in *A. pallida* some new ectodermal cells may migrate to the gastrodermis to participate in gastrodermal regeneration after bleaching.

The origin of new cells in the gastrodermis and ectodermis has yet to be identified. Since a short time of incubation in EdU-containing sea water suffices to label cells in both tissue types, it seems likely that cells are produced locally by division of either precursor cells (i.e. cells committed to a lineage) or multipotent stem cells (i.e. interstitial stem cells). Such multipotent stem cells located in the mesoglea have previously been reported during regeneration of damaged tissue [70]–[72]. New cells could also differentiate from interstitial stem cells. These cells are located under the ectodermal surface between epithelia-muscular cells and are known to be stem cells producing gametes and other phenotypes, including secretory cells [71]. While EdU+ cells are most likely produced locally, the origin of mature mucocytes remains to be solved. Transdifferentiation and migration of precursor cells from another part of the anemone, such as the tentacle basis or the oral region, cannot be excluded. Further investigation with shorter incubation periods conducted during and directly after the stress treatment will help clarify the origin of proliferating cells as well as mucocytes.

Finally, additional studies are needed to elucidate the trigger of cell proliferation (the induced stress or a consequence of a reduction in *Symbiodinium* density) as well as the nature of the signal itself. The latter question applies to the ectodermis specifically, which is not directly affected by the effects of algal loss.

In conclusion, cell proliferation in both the gastrodermis and ectodermis occurs rapidly and transiently after a combination of hyperthermia and high irradiance in the sea anemone *A. pallida*. In the gastrodermis, cell proliferation likely contributes to the regeneration of bleached tissue and provides new host cells for *Symbiodinium*. In the ectodermis, increased cell proliferation can contribute to regeneration of damaged tissue as well as enhancing the heterotrophic capacities of *A. pallida*. The augmentation of mucocyte density in bleached anemones supports this hypothesis. Further analyses on the maturation and the fate of new ectodermal cells will confirm these possibilities.
